# Diagnosis of malignancies in children with musculoskeletal complaints

**DOI:** 10.1590/S1516-31802005000100005

**Published:** 2005-01-02

**Authors:** Marcela Gonçalves, Maria Teresa Ramos Ascensão Terreri, Cássia Maria Passarelli, Lupolli Barbosa, Cláudio Arnaldo Len, Lucia Lee, Maria Odete Esteves Hilário

**Keywords:** Arthralgia, Arthritis, Neoplasms, Children, Pain, Artralgia, Artrite, Neoplasias, Criança, Dor

## Abstract

**CONTEXT:**

Musculoskeletal complaints may be associated with neoplasias as an initial manifestation of the disease. When these symptoms predominate at the onset of the disease, the differential diagnosis includes several rheumatic diseases.

**OBJECTIVE:**

To assess the frequency, clinical features and types of cancer manifested in children presenting with musculoskeletal complaints over a seven-year period.

**TYPE OF STUDY:**

Retrospective.

**SETTING:**

Discipline of Allergy, Clinical Immunology and Rheumatology, Universidade Federal de São Paulo — Escola Paulista de Medicina.

**METHODS:**

The medical records of patients with musculoskeletal complaints and final diagnosis of malignant disease were reviewed. The data collected were: age when symptoms initially presented, age at diagnosis, clinical features presented, laboratory findings, and the initial and final diagnoses.

**RESULTS:**

A final diagnosis of cancer was found in nine out of 3,528 patients (0.25%) whose initial symptom was musculoskeletal pain. The mean time between disease onset and final diagnosis was five months. The most common features presented were pauciarticular arthritis or arthralgia involving the large joints. Juvenile rheumatoid arthritis was the most frequent initial diagnosis, in four out of nine patients. Anemia was the most frequent initial hematological change. Six out of eight patients had an increased erythrocyte sedimentation rate. The lactate dehydrogenase level was raised in five out of eight patients. The malignancies found included acute lymphocytic leukemia, acute myeloid leukemia, lymphoma, neuroblastoma and Ewing’s sarcoma.

**DISCUSSION:**

The frequency of neoplasia in patients with musculoskeletal pain resembled reports in the literature. Consumptive symptoms were not the warning signal in most of our patients. In subsidiary tests, progressive anemia was the most common finding, although the peripheral blood cell count may continue to be normal for weeks or months after symptom onset.

**CONCLUSION:**

Malignancy always needs to be ruled out in cases of children with musculoskeletal complaints. Uncharacteristic clinical manifestations and nonspecific laboratory tests may cause difficulty in the final diagnosis, and rigorous investigation should be performed.

## RESUMO

**CONTEXTO:**

As queixas musculoesqueléticas podem estar associadas com neoplasias como uma manifestação inicial da doença em crianças. Quando estes sintomas predominam na apresentação da doença, o diagnóstico diferencial inclui várias doenças reumáticas.

**OBJETIVOS:**

Avaliar a freqüência, as características clínicas e os tipos de neoplasias encontrados em crianças encaminhadas ao ambulatório de reumatologia pediátrica com queixas musculoesqueléticas, no período de sete anos.

**TIPO DE ESTUDO:**

Retrospectivo.

**LOCAL:**

Disciplina de Alergia, Imunologia Clínica e Reumatologia, Departamento de Pediatria, Universidade Federal de São Paulo — Escola Paulista de Medicina, São Paulo, Brasil.

**MÉTODOS:**

Num estudo terciário, foram revisados retrospectivamente os prontuários de crianças com queixas musculoesqueléticas atendidas no ambulatório de reumatologia pediátrica cujo diagnóstico definitivo foi neoplasia. Foram avaliados: idade de início dos sintomas, idade ao diagnóstico da neoplasia, sintomas e sinais iniciais, exames laboratoriais e diagnósticos inicial e definitivo (tipo de neoplasia).

**RESULTADOS:**

O diagnóstico definitivo de neoplasia foi encontrado em nove de 3528 pacientes (0,25%) atendidos em nosso ambulatório com queixas musculoesqueléticas. O intervalo médio de tempo entre os sintomas iniciais e o diagnóstico definitivo foi de cinco meses (variação de dois a 18 meses). A apresentação inicial mais freqüente foi artrite e/ou artralgia, de caráter pauciarticular, envolvendo grandes articulações. Artrite reumatóide juvenil foi o diagnóstico inicial em 44,4% dos pacientes (4/9). Na avaliação hematológica inicial, anemia foi a alteração mais freqüente, apenas um paciente apresentou leucocitose e em nenhum paciente foi observada leucopenia. Seis de oito pacientes apresentaram velocidade de hemossedimentação elevada. A desidrogenase láctica estava aumentada em cinco de oito pacientes (63%). Para o diagnóstico definitivo foi necessária a realização de mielograma e/ou biópsia. Os tipos de neoplasias encontrados incluíram leucemia linfóide aguda, leucemia mielóide aguda, linfoma não-Hodgkin, neuroblastoma e tumor de Ewing.

**DISCUSSÃO:**

Neste estudo, a freqüência de neoplasia em pacientes com dor musculoesquelética foi semelhante ao relatado na literatura. Sintomas consumptivos não foram um sinal de alerta predominante nos nossos casos. Em relação aos exames laboratoriais, a anemia progressiva foi a alteração mais comum, embora o hemograma possa permanecer normal por semanas ou meses após o início dos sintomas.

**CONCLUSÃO:**

As neoplasias têm sempre que ser lembradas em crianças com queixas musculoesqueléticas. As manifestações clínicas incaracterísticas e os exames laboratoriais iniciais inespecíficos podem dificultar o diagnóstico definitivo e, portanto, acompanhamento rigoroso e investigações adicionais devem ser realizados.

## INTRODUCTION

Musculoskeletal complaints may occasionally be associated with neoplasias as an initial manifestation of the disease. This often leads to the referral of these patients to different specialists, thus prolonging the time between the onset of symptoms and the definitive diagnosis.

The musculoskeletal manifestations that have been associated with neoplasias include diffuse bone pain, arthritis, arthralgia and myalgia. The characteristics of the pain are useful for directing the investigator to the correct diagnosis. In lymphoproliferative diseases, bone pain is initially reported as intermittent (especially in the region of the metaphysis), and progresses to a continuous, intense and mainly nocturnal pain.^1^ In contrast, the pain relating to rheumatic diseases has low to moderate intensity, occurs mainly in the morning and is accompanied by a characteristic stiffness. Musculoskeletal complaints associated with neoplasia are due to the infiltration of joints or muscles, to intra or periarticular hemorrhage, or to paraneoplastic effects mediated by humoral factors. When these symptoms predominate at the onset of the disease, the differential diagnosis includes juvenile rheumatoid arthritis, rheumatic fever, systemic lupus erythematosus, and septic or reactive arthritis.^2^

The frequency of neoplasias in children with musculoskeletal pain ranges from 0.3 to 1%, and acute lymphoblastic leukemia is the predominant one.^2,3^ Several studies concerning the musculoskeletal manifestations of childhood leukemias have shown the importance of these complaints in the clinical presentation and follow-up of these patients.^1,4-10^

The aims of the present study were to determine retrospectively the frequency and type of occult neoplasias in children who initially presented with musculoskeletal complaints, as well as determining the clinical features and laboratory alterations that can suggest such malignancy.

## PATIENTS AND METHODS

A total of 3,528 patients with musculoskeletal complaints were referred to the Pediatric Rheumatology Outpatient Clinic of our public institution during the period from February 1994 to May 2001 and were retrospectively analyzed. The patients whose final diagnosis was neoplasia were selected and assessed in relation to the age at the onset of symptoms; age at the final diagnosis; lag time between the onset of the manifestations and the diagnosis of the neoplasia; initial signs and symptoms; laboratory tests such as complete blood count, erythrocyte sedimentation rate, C-reactive protein and lactate dehydrogenase; and also the initial and final diagnosis (type of neoplasia).

## RESULTS

Among 3,528 patients evaluated over the seven-year period, nine (0.25%) had a definitive diagnosis of neoplasia. All these nine patients complained of limb pain, arthralgia and/or arthritis.

The demographic and clinical characteristics of the patients are showed in Table 1. The mean age at the onset of symptoms was six years and five months, the mean age at the diagnosis of neoplasia was eight years, and the time elapsed until the diagnosis ranged from two to 18 months (mean of five months). Fever was the most frequently observed manifestation. Juvenile rheumatoid arthritis was the most frequent initial diagnosis (4/9), followed by reactive arthritis (2/9), rheumatic fever (1/9), juvenile dermatomyositis (1/9) and limb pain (1/9). Acute lymphocytic leukemia was diagnosed in four patients and was therefore the most frequent neoplasia, followed by acute myeloid leukemia (2/9).

The distribution of the musculoskeletal manifestations of the patients with a diagnosis of neoplasia and their forms of relief can be found in Table 1. Five patients presented arthritis, which was pauciarticular (up to four affected joints) in four patients and polyarticular (five or more affected joints) in only one patient. The most affected joints were the knees, ankles and wrists. The most frequently observed sites of arthralgia were the hips and knees.

The initial changes found upon physical examination were: hepatomegaly (4/9), splenomegaly (2/9), adenomegaly (2/9) and petechiae (1/9).

The initial laboratory tests on the patients with neoplasia are shown in Table 1. Six patients presented hemoglobin levels below 11 mg/dl; however, only two patients showed levels below 10 mg/dl. Only one patient presented leukocytosis (leukocytes > 10,000) and none had leukopenia (leukocytes < 3,500). Thrombocytopenia (platelets < 150,000) was present in three patients. Six patients showed an elevated erythrocyte sedimentation rate; lactate dehydrogenase levels were increased in five patients.

## DISCUSSION

Musculoskeletal pain is common in children, affecting 10 to 20% of those of school age. ^11,12^ Although most of these complaints are of benign origin, the diagnosis of neoplasia always needs to be ruled out. However, this may be difficult, since the characteristics of musculoskeletal pain associated with neoplasia are highly variable and nonspecific. Patients might initially present articular or extra-articular diffuse pain, while in cases of bone tumors the pain is generally localized.

Predominantly nocturnal pain is also a characteristic of benign limb pain, but it may be a warning sign of neoplasia, especially when it occurs in bone metaphysis and is continuous and intense. Some patients with neoplasias may show migratory or additive arthralgia or arthritis.

In the present study, the frequency of neoplasia in patients with musculoskeletal pain (0.25%) was similar to what has been reported in the literature.^2,3^ Cabral & Tucker^3^ analyzed 8,400 patients from two pediatric rheumatology services over a period of 14 years. Twenty-nine of them (0.3%) had neoplasia as the definitive diagnosis, and 82% of the 29 cases initially reported musculoskeletal pain. Trapani et al.^2^ found malignant disease in about 1% of 1,254 patients with musculoskeletal complaints.

Similarly to the literature,^2^ in our study acute lymphocytic leukemia was the most frequent neoplasia that presented with musculoskeletal symptoms.

In our study, the mean time between initial symptoms and the definitive diagnosis was five months, an interval similar to those reported in the literature.^2^ The longest delay in the diagnosis was observed in the case of a child with an initial diagnosis of juvenile rheumatoid arthritis, for whom the diagnosis of acute lymphocytic leukemia was only made after 18 months of specific juvenile rheumatoid arthritis therapy. This delay was due to the lack of clinical and laboratory manifestations that suggested neoplasia and to the use of steroid therapy for a short period of time.

Fever was the most important systemic manifestation and was observed in all patients. In the present study, we did not observe consumptive symptoms suggestive of neoplasia, contrary to what occurs in patients with solid tumors.

Hepatosplenomegaly and lymphadenomegaly were detected in four patients upon initial examination. These alterations are known to occur in about half of the patients as a clinical manifestation of malignant diseases, and can be observed in many patients with rheumatic diseases such as systemic lupus erythematosus and juvenile rheumatoid arthritis.^2^ Arthritis was the most frequent musculoskeletal manifestation in our study and, therefore, most of the patients were initially thought to have juvenile rheumatoid arthritis. The most frequently involved joints were the large ones, as has also been reported in the literature.^1,13,14^

Laboratory tests may be normal at the onset of the clinical manifestations of neoplasia. In the present study, blood counts indicated that anemia was the most common hematological change among the patients, although this was not serious in most cases; white blood cell and platelet changes were less frequent. Progressive anemia usually represents an early warning sign, and was found in the Trapani series.^2^

Nevertheless, it should be emphasized that in malignant diseases peripheral blood cell counts may continue to be normal for weeks or months after the onset of symptoms.^5^ Moreover, pathognomonic leukemic cells (blast cells) are generally absent at the beginning of the disease.^5,15,16^

Inflammatory markers, although nonspecific, are usually found to be increased in patients with neoplasia, as was also observed in the present study. Raised lactate dehydrogenase level has been recognized as a marker of cell turnover and has been reported to be an important test for the screening of children with a suspicion of neoplasia who initially present with musculoskeletal complaints.^17^ Wallendal et al.^17^ studied 12 patients with a final diagnosis of neoplasia whose initial manifestations were arthritis or arthralgia, and reported that all had an initial increase in the lactate dehydrogenase level. Most of our patients also presented increased lactate dehydrogenase level.

## CONCLUSION

In summary, malignant diseases should always be included in the differential diagnosis of rheumatic diseases in children who initially complain of musculoskeletal pain. Systemic manifestations such as hepatosplenomegaly, lymphadenopathy, prolonged daily fever, and pain disproportional to the clinical findings, as well as laboratory abnormalities, are suggestive of malignancy, although they may often be absent.

Early diagnosis and adequate treatment are fundamental for better prognosis and this can only be achieved if the pediatrician and rheumatologist are aware of and include malignant disease in the differential diagnosis for children with both acute or chronic limb pain, arthralgia or arthritis.

## References

[1] Costello PB, Brecher ML, Starr JI, Freeman AI, Green FA (1983). A prospective analysis of the frequency, course, and possible prognostic significance of the joint manifestations of childhood leukemia. J Rheumatol.

[2] Trapani S, Grisolia F, Simonini G, Calabri GB, Falcini F (2000). Incidence of occult cancer in children presenting with musculoskeletal symptoms: a 10-year survey in a pediatric rheumatology unit. Semin Arthritis Rheum.

[3] Cabral DA, Tucker LB (1999). Malignancies in children who initially present with rheumatic complaints. J Pediatr.

[4] Spilberg I, Meyer GJ (1972). The arthritis of leukemia. Arthritis Rheum.

[5] Ostrov BE, Goldsmith DP, Athreya BH (1993). Differentiation of systemic juvenile rheumatoid arthritis from acute leukemia near the onset of disease. J Pediatr.

[6] Jonsson OG, Sartain P, Ducore JM, Buchanan GR (1990). Bone pain as an initial symptom of childhood acute lymphoblastic leukemia: association with nearly normal hematologic indexes. J Pediatr.

[7] Saulsbury FT, Sabio H, Conrad D, Kesler RW, Levien MG (1984). Acute leukemia with features of systemic lupus erythematosus. J Pediatr.

[8] Bradlow A, Barton C (1991). Arthritic presentation of childhood leukaemia. Postgrad Med J.

[9] Fink CW, Windmiller J, Sartain P (1972). Arthritis as the presenting feature of childhood leukemia. Arthritis Rheum.

[10] Saulsbury FT, Sabio H (1985). Acute leukemia presenting as arthritis in children. Clin Pediatr (Phila).

[11] Goodman JE, McGrath PJ (1991). The epidemiology of pain in children and adolescents: a review. Pain.

[12] Malleson PN, Beauchamp RD (2001). Rheumatology: 16. Diagnosing musculoskeletal pain in children. CMAJ.

[13] Schaller J (1972). Arthritis as a presenting manifestation of malignancy in children. J Pediatr.

[14] Evans TI, Nercessian BM, Sanders KM (1994). Leukemic arthritis. Semin Arthritis Rheum.

[15] Holdrinet RS, Corstens F, van Horn JR, Bogman JJ (1989). Leukemic synovitis. Am J Med.

[16] Tuten HR, Gabos PG, Kumar SJ, Harter GD (1998). The limping child: a manifestation of acute leukemia. J Pediatr Orthop.

[17] Wallendal M, Stork L, Hollister JR (1996). The discriminating value of serum lactate dehydrogenase levels in children with malignant neoplasms presenting as joint pain. Arch Pediatr Adolesc Med.

