# Diagnosis of malignancies in children with musculoskeletal complaints

**DOI:** 10.1590/S1516-31802005000200002

**Published:** 2005-03-02

**Authors:** Marcela Gonçalves, Maria Teresa Ramos Ascensão Terreri, Cássia Maria Passarelli Lupolli Barbosa, Cláudio Arnaldo Len, Lucia Lee, Maria Odete Esteves Hilário

Due to an error by the editors, the article was printed without one of the tables (Table 1). The Table is the following. Citations to the article should use the original volume, issue, and page numbers, and cite this erratum if necessary.

**Table 1:** Distribution of children with neoplasia diagnosis attended in a public hospital in Brazil, according to the demographic, clinical and musculoskeletal characteristics and laboratory tests

Patient (gender)	1 (M)	2 (F)	3 (F)	4 (F)	5 (M)	6 (M)	7 (M)	8 (F)	9 (M)
**Age (years) at onset of symptoms**	2.5	3.9	4.2	4.5	4.9	5.8	7.0	12.2	13.8
**Constitutional symptoms**	Daily fever	Daily fever, asthenia, weight loss	Daily fever, asthenia	Daily fever	Daily fever, anorexia	Sporadic fever	Sporadic fever	Daily fever, anorexia	Daily fever
**Initial diagnosis**	Reactive arthritis	Juvenile rheuma-toid arthritis	Reactive arthritis	Juvenile der-matomyositis	Juvenile rheumatoid arthritis	Rheumatic fever	Limb pain	Juvenile rheumatoid arthritis	Juvenile rheu-matoid arthritis
**Final diagnosis**	Neuroblastoma	Acute myeloid leukemia	Acute lympho-cytic leukemia	Acute lym-phocytic leukemia	Acute lympho-cytic leukemia	Acute lympho-cytic leukemia	Ewing's sarcoma	Hodgkin's lymphoma	Acute myeloid leukemia
**Months until final diagnosis**	3	3	2	6	18	5	2	2	4
**Pain**	Left hip, cervical spine	Right knee, left hip	-	Left knee, metacarpo-phalangeal, lumbar spine	Knees, lower limbs	Left thigh, left leg	Right thigh	Right hip, right thigh	Right shoulder, right wrist
**Arthritis**	-	Right wrist, right ankle, knees	Knees	Left ankle, metacarpo-phalangeal, sacroiliac	-	Left knee	-	-	Temporoman-dibular joint, hips, left wrist, left knee, right ankle
**Relief**	Nonsteroidal anti-inflamma-tory drugs	Analgesics	Spontaneous	Massage, nonsteroidal anti-inflamma-tory drugs	Spontaneous	Massage	Analgesics	Massage, analgesics	Analgesics
**Hemoglobin (g/dl)**	10.7	6.4	9.5	10.6	10.3	10.8	12.0	11.3	12.0
**Leukocytes (n/mm^3^)**	5,100	19,000	4,300	3,700	9,300	6,100	7,500	8,600	3,600
**Platelets (p/mm^3^)**	272,000	130,000	108,000	126,000	-	166,000	212,000	450,000	165,000
**Erythrocyte sedimentation rate (mm/h)**	68	72	103	91	-	33	14	5	33
**C-reactive protein (mg/dl)**	Positive	Negative	12	316	Negative	-	Negative	Negative	24
**Lactic dehydro-genase (U/l)**	947	1,644	410	2,330	-	607	410	461	325

*F = female; M = male. Normal values for laboratory tests = hemoglobin > 11 g/dl; leukocytes > 3,500 and < 10,000/mm^3^; platelets > 150,000/mm^3^; erythrocyte sedi-mentation rate < 20 mm/h; C-reactive protein > 7 mg/dl and lactic dehydrogenase > 460 U/l.*

